# *Staphylococcus argenteus* from rabbits in Thailand

**DOI:** 10.1002/mbo3.665

**Published:** 2018-06-21

**Authors:** Nitaya Indrawattana, Natapol Pumipuntu, Nawarat Suriyakhun, Arunee Jangsangthong, Suphang Kulpeanprasit, Narisara Chantratita, Nitat Sookrung, Wanpen Chaicumpa, Shutipen Buranasinsup

**Affiliations:** ^1^ Department of Microbiology and Immunology Faculty of Tropical Medicine Mahidol University Bangkok Thailand; ^2^ Office of Academic Affairs Faculty of Veterinary Sciences Mahasarakham University Maha Sarakham Thailand; ^3^ Prasu‐Arthorn Animal Hospital Faculty of Veterinary Science Mahidol University Nakhon Pathom Thailand; ^4^ Department of Pre‐Clinic and Applied Animal Science Faculty of Veterinary Science Mahidol University Nakornpathom Thailand; ^5^ Laboratory for Research and Technology Development Faculty of Medicine Siriraj Hospital Mahidol University Bangkok Thailand; ^6^ Center of Excellence on Therapeutic Proteins and Antibody Engineering Department of Parasitology Faculty of Medicine Siriraj Hospital Mahidol University Bangkok Thailand

**Keywords:** antimicrobial resistance, antimicrobial resistance genes, rabbit, *Staphylococcus argenteus*, *Staphylococcus aureus*, *Staphylococcus aureus* complex

## Abstract

*Staphylococcus argenteus*, a novel species of the genus *Staphylococcus* or a member of the *S. aureus* complex, is closely related to *S. aureus* and is usually misidentified. In this study, the presence of *S. argenteus* in isolated *S. aureus* was investigated in 67 rabbits with abscess lesions during 2014–2016. Among 19 *S. aureus* complex isolates, three were confirmed to be *S. argenteus* by matrix‐assisted laser desorption/ionization time‐of‐flight mass spectrometry, nonribosomal peptide synthetase gene amplification, and multilocus sequence type. All *S. aureus* complex isolates, including the *S. aureus* isolates, were examined for their antimicrobial resistance phenotype by disk diffusion and for their resistance genotype by PCR assays. Among the *S. argenteus* isolates, one was susceptible to all antimicrobial drugs and the other two were resistant to penicillin and doxycycline. In contrast, most *S. aureus* isolates were resistant to penicillin (37.5%), and gentamicin (12.5%). Moreover, *S. aureus* isolates harbored the *bla*Z, *mec*A, *aac*A*‐aph*D, and *mrs*(A) as well as mutations of *gyr*A and *grl*A, but *S. argenteus* isolates carried solely the *bla*Z. *S. argenteus* isolates were investigated for enterotoxin (*sea*‐*sed*) and virulence genes by PCR. One isolate carried *sea*,* sec,* and *sed*, whereas the other two isolates carried only *sea* or *sed*. No isolate carried *seb* and *see*. All three *S. argenteus* isolates carried *hla*,* hlb,* and *clf*A, followed by *pvl*, whereas *coa*,* spa* (IgG‐binding region), and *spa* (x region) were not detected in the three isolates. This paper presents the first identification of *S. argenteus* from rabbits in Thailand. *S. argenteus* might be pathogenic because the isolates carried virulence genes. Moreover, antimicrobial resistance was observed. Investigations of this new bacterial species should be conducted in other animal species as well as in humans.

## 1 INTRODUCTION

The *Staphylococcus aureus* complex consists of opportunistic pathogens that can cause a wide spectrum of diseases in both humans and animals (Corpa et al., 2009). These pathogens are nonspore‐forming, nonmotile, spherical organisms, appearing as grapelike clusters under a microscope. They are facultatively anaerobic, catalase‐positive (Foster & Geoghegan, 2015), coagulase‐positive and can produce protein A. In rabbits, infection with the *S. aureus* complex usually results in small dermal lesions; the invasion of subcutaneous tissue and the development of pododermatitis, subcutaneous abscesses, and mastitis. Abscesses in internal organs are sometimes observed, such as in the lungs, liver, and uterus. This gives rise to poor reproductive results, infertility, and death (Corpa et al., 2009; Vancraeynest et al., 2004; Viana et al., 2007).

Recently, a novel coagulase‐positive *Staphylococcus* species, *S. argenteus* (*S. aureus* complex), was identified from clinical human and animal sources (Argudín et al., 2016; Chantratita et al., 2016; Schuster et al., 2017; Thaipadungpanit et al., 2015; Tong et al., 2015). Bacterial colonies were characterised by a nonpigmented, creamy white appearance and showed β‐hemolysis on blood agar. Moreover, the bacteria were shown to be gram‐positive cocci in clusters and gave positive results in the catalase and coagulase tests, which are characteristic findings for *S. aureus* (Tong et al., 2015). Therefore, routine diagnostic analyses can lead to *S. argenteu*s being misidentified as *S. aureus*. Moreover, identification by molecular methods, such as 16S rRNA sequencing, cannot differentiate *S. argenteus* from *S. aureus* (Tong et al., 2015). Other molecular techniques, such as matrix‐assisted laser desorption/ionization time‐of‐flight mass spectrometry (MALDI‐TOF MS), nonribosomal peptide synthetase (NRPS) gene amplification, and multilocus sequence type (MLST) determination, were thus recommended in many publications as tools for the identification of *S. argenteus* (Chantratita et al., 2016; Schuster et al., 2017; Zhang et al., 2016). Some sequencing types of *S. aureus* were previously confirmed to be *S. argenteus*, such as ST2793, ST1223, and ST2250 (Chantratita et al., 2016; Schuster et al., 2017; Thaipadungpanit et al., 2015; Tong et al., 2015).

The aim of this study was to characterize *S. argenteus* and *S. aureus* isolated from rabbits with clinical abscesses.

## 2 MATERIALS AND METHODS

### 2.1 Specimen collection and bacterial isolation

Sixty‐seven pus samples were collected from rabbits with clinical abscesses by a veterinarian at Prasu‐Arthorn Animal Hospital, Thailand, during 2014–2016. They were transported to a microbiological laboratory within 24 hr after collection. Individual samples were inoculated on sheep blood and mannitol salt agar (Oxoid, Basingstoke, UK) and incubated at 37°C for 24–48 hr. After incubation, the suspected bacterial colonies were selected and identified by conventional methods, including Gram staining, catalase, mannitol fermentation, coagulase (Ramel; Oxoid), and commercial latex agglutination (Dryspot Staphytect Plus; Oxoid), to detect protein A for *S. aureus* identification. This study was approved by The Faculty of Veterinary Science Animal Care and Use Committee, Mahidol University (protocol number MUVS‐2013‐35).

### 2.2 *S. argenteus* identification

#### 2.2.1 MALDI‐TOF MS

Mass spectra were generated using a MALDI Biotyper 3.0 Ultraflex platform (Bruker Daltonics, Massachusetts, USA). For individual suspected *S. argenteus* isolates, with white colonies, 1 ml of crude protein extract or one colony was deposited on a 96‐spot polished steel target plate (Bruker Daltonics), air‐dried and covered with 1 ml of HCCA matrix solution (Bruker Daltonics) (Kolecka et al., 2013). As a positive control and calibration reference, 1 ml of Bacterial Test Standard (Bruker Daltonics) was used. The main spectrum was acquired using the MALDI Biotyper Automated Flex Control software v.3.0 (Bruker Daltonics). The identification of isolates was performed using the Bruker database and in‐house databases from Chantratita et al. (2016) and Moradigaravand et al. (2017).

#### 2.2.2 NRPS gene amplification

Primer sequences and the PCR protocol for NRPS gene indels were in accordance with those of Zhang et al. (2016). The NRPS gene was amplified in a total reaction volume of 25 μl. The PCR reaction was performed using the thermal cycles (Bio‐Rad, California, USA) with initial denaturation at 94°C for 4 min, followed by 35 cycles of 94°C for 30 s, 53°C for 30 s, and 72°C for 40 s and then final extension at 72°C for 10 min. Individual PCR amplicons were purified using the GenepHlow^™^ Gel/PCR purification kit (Geneaid, New Taipei, Taiwan) and submitted for sequencing. DNA sequences were analyzed for similarity with the GenBank database.

#### 2.2.3 MLST

MLST was used to analyze the suspected *S. argenteus* isolates through the amplification of seven *S. aureus* housekeeping genes, by a method developed by Enright *et al*. (2000) at Imperial College London for analyzing a query profile for MLST. Before sequencing, individual PCR amplicons were purified using the GenepHlow^™^ Gel/PCR purification kit (Geneaid). The allelic number queries and sequence types (STs) obtained from trimmed DNA sequencing results for seven genes were determined using the online *S. aureus* MLST database (https://pubmlst.org/saureus/). The suspected novel alleles or queried allelic profiles of novel STs that did not match the database were submitted to the curator of PubMLST (https://pubmlst.org/saureus/) to check and assign a novel allele or novel ST number.

### 2.3 Antimicrobial susceptibility testing

Antimicrobial susceptibility testing and interpretation were performed by the disk diffusion in according to the Clinical and Laboratory Standards Institute (2012). A total of 13 antimicrobial drugs were tested: amikacin (30 μg), azithromycin (15 μg), cefazolin (30 μg), cefoxitin (30 μg), ceftriaxone (30 μg), chloramphenicol (30 μg), ciprofloxacin (5 μg), doxycycline (30 μg), gentamicin (10 μg), moxifloxacin (5 μg), norfloxacin (10 μg), penicillin (10 units), and trimethoprim/sulfamethoxazole (1.25 μg/23.75 μg). *S. aureus* ATTC^®^25923 was used as the control strain.

### 2.4 Detection of 16s rRNA, antimicrobial resistance, and enterotoxin and virulence genes

All isolated *S. aureus* samples were prepared for genomic DNA extraction using a DNA extraction kit (Geneaid). The extraction protocol involved the measurement of the OD 260/280 nm ratio using a spectrophotometer. Amplification with specific oligonucleotide primers was performed for 16s rRNA of the *S. aureus* complex group (McClure et al., 2006) and eight antimicrobial resistance genes representative of each antibiotic drug group: *bla*Z (Gómez‐Sanz et al., 2010) and *mec*A (Vancraeynest et al., 2004) for β‐lactam resistance, *aac*A*‐aph*D (Laplana et al., 2007) for aminoglycoside resistance, *mrs*(A) (Gómez‐Sanz et al., 2010) for macrolide resistance, *tet*(K) (Vancraeynest et al., 2004) for tetracycline resistance, *dfr*G (Lagier et al., 2009) for trimethoprim resistance, and *cfr* (Kehrenberg & Schwarz, 2006) for chloramphenicol resistance. PCR amplification for determined fluoroquinolone resistance, namely *gyrA* and *grlA* (Iihara et al., 2006) was performed. The PCR amplicon, namely the QRDR region, was sequenced and analyzed for resistance determining mutations. Deduced amino acid sequences of the PCR amplicons were analyzed using the GenBank database with accession numbers AAC31138.1 for *gyr*A and WP075108737.1 for *grl*A. The PCR reaction mixture was subjected to the following thermal cycling conditions using Flexcycler^2^ (Analytik Jena, Überlingen, Germany): 5 min of 95°C; then 30 cycles of amplification with denaturing at 95°C for 30 s, annealing at a temperature specific for each primer for 30 s and extension at 72°C for 60 s; followed by a final extension at 72°C for 10 min.

In *S. argenteus* isolates, further identification of the virulence genes was performed, including classical enterotoxin (*sea*,* seb*,* sec*,* sed,* and *see*) (Wu et al., 2011), hemolysin (*hla* and *hlb*) (Jarraud et al., 2002), clumping factor (*clf*A) (Tristan et al., 2003), protein A [*spa* x region (Frénay et al., 1996) and *spa* IgG‐binding region (Seki et al., 1998)], coagulase (*coa*) (Aslantas et al., 2007), and Panton‐Valentine leukocidin (*pvl*) (Jarraud et al., 2002), with specific oligonucleotide primers. PCR reactions were performed, involving initial denaturation at 95°C for 10 min; 35 cycles of 95°C for 30 s, 55°C for 30 s, and 72°C for 30 s and a final extension at 72°C for 10 min.

PCR amplicons were analyzed using 1.5% agarose gel electrophoresis and SYBR safe (Invitrogen, New York, USA) staining. The DNA bands were observed under a UV transilluminator (UVP Bioimaging System; Invitrogen). Control bacteria for the PCR included the strains *S. aureus* ATCC 19095 (*sea* and *sec*), ATCC 14458 (*seb*), ATCC 23235 (*sed*), ATCC 27664 (*see*), ATCC 13565 (*coa*,* clf*A, *hla*,* hlb*,* spa* x region, and *spa* IgG‐binding region) and laboratory control strains, which were sequenced and analyzed as accession numbers KX371630.1 (*pvl*). For gene amplification with no reference control, the PCR product from positive samples was subjected to nucleotide sequencing and sequence analysis for gene confirmation.

## 3 RESULTS

### 3.1 Detection of *S. argenteus* by MALDI‐TOF MS

From 67 samples (total of 19 bacterial isolates), we obtained 11 *S. aureus* isolates and 8 suspected *S. argenteus* isolates. These suspected *S. argenteus* isolates were analyzed by MALDI‐TOF MS. After visual inspection and obtaining the spectral results of their ionizable cell surface components, which were compared for similarity with the spectral data in the Bruker and in‐house databases. The results showed that there are five isolates that match with the *S. aureus* database and three isolates that match with the *S. argenteus* database, with a high score (>2.3) (isolate no. U19 T10.1, U43 S18.1, and U43 S18.3; Table 1).

**Table 1:** Prevalence of enterotoxin and virulence genes, MLST and MALDI‐TOF MS for suspected *Staphylococcus argenteus* identification

Isolate no.	Enterotoxin and virulence genes detection	MLST identification		Sequence results of NRPS PCR amplicon			MALDI‐TOF MS identification
		ST	CC	Size (bp)	BLAST species	Sequence identities (%)	
U27 OC2.1	ND	ST4209^a^	Singleton	160	*S. aureus* strain K5 (CP020656.1)	97	*S. aureus*
U27 OC2.2	ND	ST4209^a^	Singleton	160	*S. aureus* strain FDAARGOS (CP007539.3)	99	*S. aureus*
U27 OC2.3	ND	ST4209^a^	Singleton	160	*S. aureus* strain K5 (CP020656.1)	96	*S. aureus*
U19 T10.1	*sed, hla, hlb, clf*A*, pvl*	ST4210^a^, ^b^	Singleton	340	*S. argenteus* strain XNO106 (CP025023.1)	99	*S. argenteus*
U43 S18.1	*sea, hla, hlb, clf*A	ST4211^a^, ^b^	Singleton	340	*S. argenteus* strain XNO106 (CP025023.1)	100	*S. argenteus*
U43 S18.3	*sea, sec, sed, hla, hlb, clf*A*, pvl*	ST4211^a^, ^b^	Singleton	340	*S. argenteus* strain XNO106 (CP025023.1)	100	*S. argenteus*
U14 T6.2	ND	ST4212^a^	Singleton	160	*S. aureus* strain FDAARGOS_159 (CP014064.2)	95	*S. aureus*
U65 S3	ND	ST4213^a^	Singleton	160	*S. aureus* strain NRS137 (CP026080.1)	92	*S. aureus*

*Note*

bp: base pair; BLAST: basic local alignment search tool; CC: clonal complex; MALDI‐TOF MS: matrix‐assisted laser desorption/ionization time‐of‐flight mass spectrometry; ND: not determine; MLST: multilocus sequence type; NRPS: nonribosomal peptide synthetase.

a Novel STs from this study.

b *S. argenteus* ST.

### 3.2 NRPS gene amplification of *S. argenteus*

**Figure 1:**
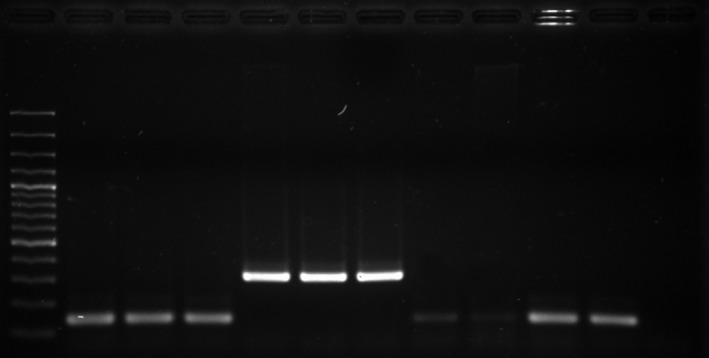
Non ribosomal peptide synthetase amplicon of eight suspected *Staphylococcus argenteus*. Lane M: DNA marker; lane 1: isolate no. U27 OC2.1; lane 2: isolate no. U27 OC2.2; lane 3: isolate no. U27 OC2.3; lane 4: isolate no. U19 T10.1; lane 5: isolate no. U43 S18.1; lane 6: isolate no. U43 S18.3; lane 7: isolate no. U14 T6.2; lane 8: isolate no. U65 S3; lane 9: *S. aureus* ATCC13565; lane 10: *S. aureus* ATCC25923; lane 11: negative control

The NRPS gene was amplified for eight suspected *S. argenteus* isolates, as shown in Figure 1. The results showed that three isolates (U19 T10.1, U43 S18.1, and U43 S18.3) have a PCR product of approximately 340 bp, which was correlated with *S. argenteus* as reported by Zhang et al. (2016). In contrast, the five other suspected isolates had a PCR product of nearly 160 bp, which was reported to correlate with *S. aureus* (Zhang et al., 2016). The sequences of NRPS amplicons were analyzed for similarity using the GenBank database, which showed that bacterial isolate no. U19 T10.1, U43 S18.1, and U43 S18.3 have 99% identity with *S. argenteus* strain XNO106 (accession number: CP025023.1). In contrast, the other five isolates showed high identity with *S. aureus* strain K5 (accession number: CP020656.1), *S. aureus* strain FDAARGOS (accession number: CP007539.3), *S. aureus* strain FDAARGOS_159 (accession number: CP014064.2), and *S. aureus* strain NRS137 (accession number: CP026080.1). These results correlated with the MALDI‐TOF MS results (Table 1).

### 3.3 MLST of *S. argenteus*

**Figure 2:**
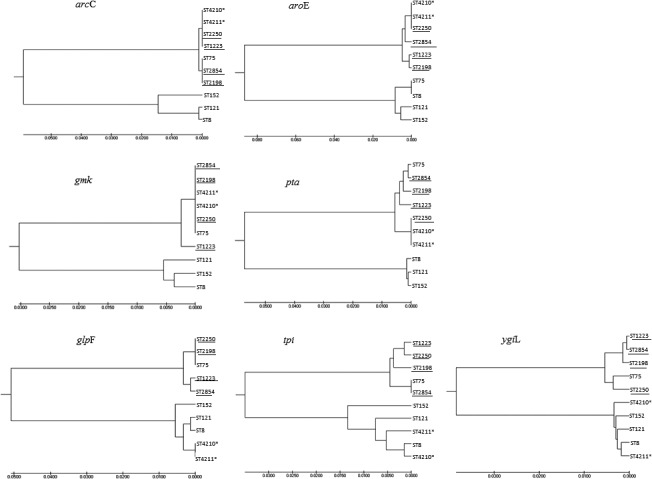
Phylogenetic neighbor‐joining tree of suspected *Staphylococcus argenteus*. The tree is constructed from *arcC*,* aroE*,* gmk*,* glpF*,* pta*,* tpi,* and *ygiL* gene sequences from the suspected *S. argenteus* (ST4210*, ST4211*), *S. aureus* reference strain (ST75, ST152, ST121, ST8) and ST of published *S. argenteus* group (ST1223, ST2250, ST2854, ST2198). The phylogenetic analysis was performed using MEGA7

All eight suspected *S. argenteus* isolates were further analyzed by MLST to define STs. Several isolates were shown to be novel STs of the *Staphylococcus* complex, which included ST4209 (isolate no. U27 OC2.1, U27 OC2.2, and U27 OC2.3), ST4210 (isolate no. U19 T10.1), ST4211 (isolate no. U43 S18.1 and U43 S18.3), ST4212 (isolate no. U14 T6.2), and ST4213 (isolate no. U65 S3), derived from the curator of the PubMLST *S. aureus* database (https://pubmlst.org), University of Oxford, UK, and the Wellcome Trust fund. The results showed that three isolates (isolate no. U19 T10.1, ST4210; isolates no. U43 S18.1 and U43 S18.3, ST4211) were identified as *S. argenteus*, which correlated with the results of MALDI‐TOF MS and the NRPS gene. The neighbor‐joining and maximum likelihood analyses yielded similar phylogenetic trees. Based on *arc*C, *aro*E, *gmk,* and *pta*, three bacterial isolates, ST4210 (isolate no. U19 T10.1) and ST4211 (isolates no. U43 S18.1 and U43 S18.3), showed close similarity to the *S. argenteus* group (ST1223, ST2250, ST2854, and ST2198) (Figure 2).

### 3.4 Enterotoxin and virulence genes

Five classical enterotoxin genes and seven virulence genes, as mentioned in the Materials and Methods section, were investigated in the three *S. argenteus* isolates by PCR, the results of which are shown in Table 1. The detected enterotoxin genes were *sea*,* sec,* and *sed*. No isolate carried *seb* and *see*. Regarding the virulence genes, the most prevalent ones were *hla*,* hlb,* and *clf*A present at a rate of 100%, followed by *pvl* at 66.67%, whereas *coa*,* spa* (IgG‐binding region) and *spa* (x region) were not detected in the *S. argenteus* isolates.

### 3.5 Antimicrobial susceptibility testing

From bacterial identification, we obtained 16 *S. aureus* isolates and three *S. argenteus* isolates. Among the three *S. argenteus* isolates, one (isolate no. U19 T10.1) was susceptible to all the tested antimicrobial drugs, whereas the remaining two (isolate no. U43 S18.1 and U43 S18.3) were resistant to penicillin and doxycycline. Meanwhile, among the *S. aureus* isolates, six were resistant to penicillin and two were resistant to gentamicin. One *S. aureus* isolate (isolate no. U14 T6.2) was resistant to several antimicrobial classes, namely, β‐lactams, aminoglycosides, macrolides, tetracyclines, and fluoroquinolones.

### 3.6 Antimicrobial resistance genes

In the *S. aureus* and *S. argenteus* isolates, the presence of antimicrobial resistance genes was tested. We found that all *S. aureus* isolates carried *bla*Z, *mec*A, *aac*A*‐aph*D, and *mrs*(A), but none carried *dfr*G, *tet*(K), and *cfr*, whereas all three *S. argenteus* isolates carried only the *bla*Z (isolate no. U43 S18.1 and U43 S18.3). Mutations of *gyr*A and *grl*A were found in the *S. aureus* isolate no. U14 T6.2. The mutations of *gyr*A occurred at codons 88 [GAA (Glu) → GAT (Asn)] and 96 [GAT (Asp) → ACA (Thr)], whereas the mutation of *grl*A occurred at codon 80 [TCC (Ser) → TTA (Leu)]. The presence of antimicrobial resistance genes in *S. aureus* is shown in Table 2.

**Table 2:** Antimicrobial drug resistance phenotypes and antimicrobial resistance genes of *Staphylococcus aureus* isolated from rabbit

Antimicrobial susceptibility testing				Antimicrobial resistance genes amplification	
Drug	S (%)	I (%)	R (%)	Gene	No. of isolates (%)
β‐lactams					
Penicillin	62.5	0	37.5	*bla*Z	1 (6.25)
Cefoxitin	93.75	0	6.25	*mec*A	1 (6.25)
Cefazolin	93.75	0	6.25		
Ceftriaxone	75.0	18.75	6.25		
Aminoglycosides					
Gentamicin	87.5	0	12.5	*aac*A*‐*	1 (6.25)
Amikacin	93.75	0	6.25	*aph*D	
Macrolides					
Azithromycin	93.75	0	6.25	*mrs*(A)	1 (6.25)
Tetracycline					
Doxycycline	93.75	0	6.25	*tet*(K)	0 (0)
Fluoroquinolones					
Ciprofloxacin	93.75	0	6.25	*gyr*A	1 (6.25)
Moxifloxacin	93.75	0	6.25	*grl*A	1 (6.25)
Norfloxacin	93.75	0	6.25		
Folate pathway inhibitors					
Trimethoprim/sulfamethoxazole	100	0	0	*dfr*G	0 (0)
Phenicols					
Chloramphenicol	100	0	0	*cfr*	0 (0)

*Note*

S: susceptible; I: intermediate; R: resistance.

## 4 DISCUSSION

Staphylococci are considered the most important veterinary bacterial pathogens because they cause a multiplicity of infections and a wide range of diseases in many host species, including humans and animals (Holmes et al., 2016). This virulent group of pathogens is not only important for livestock, causing conditions such as bovine mastitis or lameness in chickens, but also for causing skin infections resulting in abscesses in pets, such as dogs, cats, and rabbits (Drougka et al., 2016; Goñi et al., 2004; Loncaric et al., 2014; Youn et al., 2014). Pathogenic strains of staphylococci that cause skin infections have been well studied and characterized. Moreover, in farm rabbits, abscesses in the lungs, liver, and uterus lead to poor production, infertility, and death (Corpa et al., 2009). This study revealed the presence of *S. aureus*, including a new member of the *S. aureus* complex, *S. argenteus*, which caused skin infections producing abscesses in rabbits.

Intriguingly, from the 19 *S. aureus* isolates, eight had white colonies, which differs from the normal colony color of *S. aureus*, which is golden or yellowish. The suspected eight isolates were further analyzed using MALDI‐TOF MS, and bacterial diversity discriminated by NRPS amplification and MLST. The MALDI‐TOF MS results revealed three *S. argenteus* isolates, which were investigated for enterotoxin and virulence genes. The enterotoxins detected were *sea* and *sed*. All three isolates carried the common virulence *hla*,* hlb,* and *clf*A. Surprisingly, previous studies have reported that *S. argenteus* is negative for the *pvl* (Thaipadungpanit et al., 2015), but we found the *pvl* in two *S. argenteus* isolates in this study. These three *S. argenteus* isolates, isolated from rabbits, had the ability to cause severe illness in these animals, particularly via the presence of *clf*A, which usually contributes to abscess formation in rabbits, as previously reported (Malachowa et al., 2016). Although *S. argenteus* is regionally distributed in animals other than humans, they have been misidentified as *S. aureus*; this has been suggested in several previous publications (Chantratita et al., 2016; Schuster et al., 2017; Thaipadungpanit et al., 2015; Tong et al., 2015).

The NRPS gene has been identified in studies of a diverse array of related *S. aureus* and *S. argenteus*. This study found that NRPS amplification can differentiate *S. argenteus* from *S. aureus*, as also reported previously (Zhang et al., 2016). The sequences of NRPS amplicons analyzed using the GenBank database also corresponded to the MALDI‐TOF MS results. Furthermore, when we performed molecular identification using MLST, all eight isolates were found to have diverse novel STs belonging to *S. aureus* (ST4209, ST4212, and ST4213) and *S. argenteus* (ST4210 and ST4211). The results showed high heterogeneity among these pathogenic bacterial isolates from rabbits. Moreover, this study revealed a new pathogenic member of the *S. aureus* complex, *S. argenteus*, for the first time. These bacteria form a genetically diverse lineage from *S. aureus* (Tong et al., 2015), being recently discovered in humans, in 2014. This study may be the first to report on *S. argenteus* originating from rabbits. We found that one *S. argenteus* isolate was susceptible to all the tested antibiotic agents and the other two *S. argenteus* isolates were resistant to penicillin and doxycycline. These results could be useful for veterinarians who have difficulty treating rabbits successfully with penicillin. In this study, we found one *S. aureus* isolate with mutations of *gyr*A and *grl*A, which indicated the possibility of quinolone resistance. Mutations in these genes have typically been found at codon 88 [GAA (Glu) → AAA (Lys)] in *gyr*A (Griggs et al., 2003; Iihara et al., 2006) and codon 80 [TCC (Ser) → TTC (Phe)] in *grl*A (Aligholi et al., 2011; Iihara et al., 2006). Interestingly, the mutation of *grl*A at codon 80 found in our study was TCC (Ser) → TTA (Leu), and the mutation of *gyr*A at codon 88 was GAA (Glu) → GAT (Asp). Comparing the antibiotic resistance pattern, the isolated *S. argenteus* showed higher susceptibility to antibiotic agents than the isolated *S. aureus*. However, it is necessary to monitor the development of drug resistance in *S. argenteus* in the future. From the discovery of *S. argenteus* in rabbits, further study of its virulence factors, pathogenesis, clinical manifestations, antimicrobial resistance, and severity or outcome should be performed to improve our knowledge for treating, controlling, or preventing this novel pathogen in exotic pets.

## CONFLICT OF INTEREST

The authors state that there are no conflicts of interest.
